# Unmet needs in the management of migraine in Greece from the perspective of medical experts: a Delphi consensus

**DOI:** 10.3389/fneur.2025.1556808

**Published:** 2025-02-12

**Authors:** Georgia Kourlaba, Michail Vikelis, Theodoros Karapanayiotides, Argyro Solakidi, Dimitrios Trafalis, Katerina Lioliou, Panagiotis Andriopoulos, Aspasia Panagiotou, Dimos-Dimitrios Mitsikostas

**Affiliations:** ^1^Department of Nursing, Faculty of Health Sciences, University of the Peloponnese, Tripoli, Greece; ^2^Headache Clinic, Mediterraneo Hospital, Glyfada, Greece; ^3^2nd Department of Neurology, Faculty of Health Sciences, School of Medicine, AHEPA University Hospital, Aristotle University of Thessaloniki, Thessaloniki, Greece; ^4^Pfizer Hellas, Athens, Greece; ^5^1st Neurology Department, Eginition Hospital, Medical School, National and Kapodistrian University of Athens, Athens, Greece

**Keywords:** migraine, Delphi consensus, neurologists, burden, unmet needs, Greece

## Abstract

**Introduction:**

Migraine is a chronic, debilitating neurological disorder affecting billions worldwide. While not life-threatening, migraine patients experience significant unmet needs in diagnosis and management. Addressing these challenges could result in improvement of patient outcomes and reduction of the socioeconomic burden migraine imposes on individuals, healthcare system and the society.

**Objective:**

This survey aimed to capture in Greece the perspective of medical experts (neurologists) specializing in migraine management regarding the socioeconomic burden of migraine and the unmet needs in diagnosis and treatment.

**Methods:**

An online Delphi-based survey was conducted with 13 neurologists, experts in migraine. The survey consisted of 55 statements derived from literature research, regarding the burden of disease, diagnosis, treatment and unmet needs. Participants’ level of agreement for each statement was measured through a 5-point Likert scale (“Strongly Agree,” “Agree,” “Neither Agree nor Disagree,” “Disagree” and “Strongly Disagree”). Three rounds of voting were conducted to achieve consensus. The consensus threshold was set at 70% of responses, focusing on “Strongly Agree”/ “Agree” or “Disagree”/ “Strongly Disagree.”

**Results:**

Most experts agreed on statements emphasizing on financial and social impact of the disease and its epidemiology. However, consensus was not reached on statements concerning patient preferences for treatment administration, treatment adherence, opioid use, and frequency of visits to neurologists. Consensus was reached on the need for better healthcare professional training and the development of effective, safe treatments.

**Conclusion:**

This survey highlighted the challenges of prompt diagnosis and effective management of migraine. Addressing these needs requires patient-centered approaches, enhanced healthcare-provider training, tailored therapeutic interventions, and advanced communication platforms.

## 1 Introduction

Migraine is the most prevalent neurological disorders and one of the foremost causes of disability (1, 2). It is broadly categorized into episodic migraine-EM (less than 15 days per month, with subcategories of 4–8 days as low frequency and 8–14 days as high frequency) and chronic migraine-CM (over 15 headache days per month, of which at least eight are migraine-related or responsive to migraine-specific medication, for a period of over 3 months) (3, 4). Although the International Classification of Headache Disorders the 3rd edition (ICHD-3) formally classifies only CM, we use the terms EM and CM descriptively to reflect differences in migraine frequency and burden as is common in epidemiological research (4). The number of migraine sufferers worldwide has been estimated to 1.1 billion, with the highest prevalence observed in individuals aged 30 to 34 for both males and females (5, 6). Furthermore, migraine is 2–3 times more prevalent in females than males (6). Migraine prevalence in Greek adults up to 70 years-old is estimated at 8.0% (CM: 1.0% and EM: 7.1%) (7).

Untreated migraine usually manifests with recurrent episodes of moderate to severe headaches lasting up to 72 h, as well as nausea, vomiting, sensitivity to light and sound, and depressive symptoms (6). Recent data suggests that headache disorders (including migraine) account for the third highest burden of neurological diseases as measured by disability-adjusted life years (DALYs) in 2019 (8). There is a significant clinical burden associated with migraine because it commonly coexists with other conditions such as depression, anxiety, epilepsy, chronic pain, and cardiovascular events (9). Moreover, migraine causes considerable disruption to daily activities (10–16) and substantial healthcare costs, with indirect costs constituting approximately 90% of its overall economic burden in Europe (14, 17, 18).

Studies conducted in Greece revealed significant impact on job performance and quality of life and emphasized the increased financial burden and psychological impact of migraine on Greek patients (7, 19–26). However, despite advances in migraine treatment, patient-based surveys raise unmet medical needs concerning underdiagnosis, ineffective disease management and lack of awareness (7, 27, 28). However, it is possible that physicians’ perceptions regarding disease burden, treatment management, patient preferences, and unmet medical needs differ from those of patients (29–31). In this context, it is crucial to capture perspectives and beliefs of medical experts regarding migraine. Such information could provide useful insights for decision-makers involved in reimbursement decisions for new treatments. Additionally, it could improve physician-patient interaction, relieve the burden of migraine, and help medical experts adopt a patient-centered approach. The present study aims to document Greek neurologists’ perspectives regarding disease burden, treatment, and unmet needs of migraine.

## 2 Methods

### 2.1 Study design and participants

In this study, the Delphi technique, a widely recognized effective method for achieving maximum consensus among experts, was selected (32–36). Delphi panel, encompassing three voting rounds, was carried out from July 5 to 25, 2023. A hybrid approach was used, combining traditional and modified Delphi techniques, whereby feedback was provided to each panelist between rounds, but without the option to edit or comment on the statements. There were three distinct phases to our research. First, a questionnaire including statements extracted from the literature was developed by a Steering Committee of three neurologists-experts in migraine and an expert of research methodology. During the second phase, the participants engaged in three rounds of voting to reach a consensus. In the third and final phase, data analysis and results were reported.

Eligible to participate in our study were neurologists with expertise on migraine. Expertise was defined as having at least one PubMed publication on migraine disease and working in a headache center. A sample of 20 experts on migraine were selected by the Steering Committee in such a way as to have representation from the private sector, university hospitals and public hospitals. An invitation was sent via email to these neurologists asking them to complete and sign an informed consent form. Thirteen of them responded positively, (65% participation rate) constituting the final sample of our study. Although, there is no specific number of Delphi panel members, these studies typically involve between 10 to 20 participants, therefore the study sample was considered sufficient (35).

### 2.2 Data collection

The first phase of this study involved the creation of statements based on extensive literature research covering migraine’s epidemiology, comorbidities, guidelines in management and treatment (both national and international), financial, humanistic burden and unmet medical need. The literature search was conducted by the Steering Committee across multiple sources, including clinical guidelines, consensus and research papers, and systematic reviews published in PubMed database as well as in other scientific society websites. A total of 55 statements were included in the final questionnaire divided in six thematic sections: **Epidemiology and comorbidities** (8 Statements), **Burden of migraine** (12 Statements), **Economic burden of migraine** (4 Statements), **Diagnosis** (7 Statements), **Treatment** (15 Statements) and **Unmet need** (9 Statements). In the second phase, the questionnaire was developed in Qualtrics (Provo, United States) and distributed to participants via e-mail. To ensure anonymity, email addresses were not recorded to encourage honest responses. Participants were asked to rate their level of agreement at each statement using a 5-point Likert scale: “Strongly Agree,” “Agree,” “Neither Agree nor Disagree,” “Disagree,” and “Strongly Disagree.” Participants received periodic email reminders to enhance response rates and survey completeness. After the completion of the initial round, the data was processed, and participants received the consolidated results. A second round of voting was conducted, which included the statements that had not achieved consensus in the previous round, considering the results of the first round. After reprocessing the data from the second round, a third and final round was conducted.

### 2.3 Data management and analysis

To assess the extent of agreement or disagreement, the mean agreement level for each statement was computed based on the Likert scale in each survey round. The consensus threshold was determined based on the guidelines proposed by Hasson et al. for the Delphi technique, which recommended a level of consensus between 51 and 80% (33). Since the primary objective of this study was to identify unmet medical needs in migraine management, rather than establish guidelines, a consensus threshold of 70% was selected, focusing on borderline options (“Strongly Agree,” “Agree,” “Disagree,” “Strongly Disagree”).

### 2.4 Ethics statement

For the study protocol ethical approval was obtained from the Research Ethics Committee of the University of the Peloponnese. Each participant in the study was required to provide written informed consent after being fully informed of the study’s objectives, time commitment, and use of their personal information (33, 37). The Delphi process did not involve any anticipated risks or financial benefits. In order to maintain the confidentiality of participants and their responses in the online survey, all data collected during the study was anonymized (37). In addition, all research data has been managed in accordance with the European and national regulations that govern the protection of personal data in scientific research.

## 3 Results

### 3.1 Epidemiology and comorbidities

A consensus was reached for all eight statements considered in this section (Table 1). There was consensus (100%) in agreement among the medical experts regarding the annual global prevalence and the annual prevalence of migraine in the adult Greek population (77.8%). Unanimously, the medical experts disagreed that the prevalence has increased in the last 30 years by about 2%, while agreed that migraine is more prevalent in women particularly those under 50 years old (100%), and among individuals aged 20–35 years (100%). Additionally, the medical experts agreed that despite the increased prevalence, migraine remains an underdiagnosed disease (100%). Finally, there was 100% agreement that mood disorders, anxiety and sleep disorders often occur along with migraines, while there was consensus in disagreement regarding the claim that cardiovascular diseases frequently co-exist with migraine (72%).

**Table 1:** Results regarding epidemiology and comorbidities.

Statements	Round	Level of consensus
Migraine is a disease with an estimated annual global prevalence of 15.2% in the general population, with approximately 14.2% of the general population suffering from episodic migraine (4, 5).	1st	100% Agreement
In Greece, according to the latest data, the annual prevalence of migraine in the adult population (18–70 years old) is 8.1%, with episodic migraine estimated at 7.1% (7).	1st	77.8% Agreement
The prevalence of migraine has increased over the last three decades by about 2% (6).	3rd	72% Disagreement
Migraine is more common in women and even more in those under 50 years old (9).	1st	100% Agreement
Migraine is common in the more productive age group of 20–35 years (43, 73)	1st	100% Agreement
Despite the increase in the prevalence of migraine in recent years, it is estimated that it is still an under-diagnosed disease (48, 74).	1st	100% Agreement
Mood, anxiety and sleep disorders are often comorbidities of migraine (75–77).	1st	100% Agreement
Cardiovascular diseases are often comorbidities of migraine (78).	3rd	72% Disagreement

### 3.2 Burden of migraine

On all 12 statements in this section, consensus was reached in agreement (Table 2). According to all medical experts, migraine significantly impacts patients’ quality of life (100%), and patients’ productivity, posing a significant burden on employers, patients, and society (100%). Moreover, medical experts concluded that severe migraine attacks can confine patients to bed, preventing them from performing daily and social activities (100%). Additionally, 77.8% of medical experts concurred that patients are often absent from work during these attacks, and 100% acknowledged that migraines impact not only patients but also their families. In addition, the medical experts agreed that migraine patients under the age of 45 generally suffer from a greater burden in their social and professional lives (88.9%) and that they frequently modify and adapt their lives to avoid future episodes (88.9%). As for stigma, medical experts agreed that this is an underestimated feature experienced by patients (88.9%). Finally, medical experts pointed out that migraine patients often feel that people in their social environment as well as healthcare professionals underestimate the severity of their condition (100%).

**Table 2:** Results regarding Burden of migraine (Quality of life, social impacts, psychological impacts, Work-related impacts).

Statements	Round	Level of consensus
Migraine significantly affects the quality of life of patients (44).	1st	100% Agreement
Based on the “Years of Living with Disability Index,” migraine is ranked by the WHO as the second cause of disability among all medical conditions and syndromes (38, 45).	1st	100% Agreement
Migraine affects significantly the productivity of patients, placing a significant burden on employers, patients and society in general (10, 11, 46–53).	1st	100% Agreement
Migraine patients are often absent from work when they have migraine attacks (47).	1st	77.8% Agreement
Migraine patients experience significant problems in their social interactions during migraine attacks and also interictally (9, 44, 54).	1st	100% Agreement
Stigma is an underestimated feature of migraine experienced by migraine patients (45).	1st	88.9% Agreement
Many patients suffering from severe migraine attacks are unable to perform daily activities and may be confined to bed during a migraine attack (44).	1st	100% Agreement
Migraine patients feel that people in their social environment underestimate the severity of their problem (55).	1st	100% Agreement
Migraine patients feel that healthcare professionals do not fully understand the severity of the symptoms they are experiencing (56).	1st	100% Agreement
Migraine patients often modify and adapt their lives to avoid an upcoming episode (54).	1st	88.9% Agreement
Migraine does not only affect the patients but also their family members (79, 80).	1st	100% Agreement
Migraine patients younger than 45 years old generally have a greater burden in social and working life (19).	1st	88.9% Agreement

### 3.3 Economic burden of migraine

Consensus in agreement was reached for all four statements regarding the economic burden of migraine (Table 3). Specifically, medical experts agreed at 77.8% that migraine has a high economic burden comparable to dementia and strokes. This high economic burden is primarily attributed to the substantial loss of productivity among patients (88.9%). Also, there was consensus at 77.8% regarding the extensive use of healthcare resources by migraine patients. This involves frequent visits to doctors of varying specialties and to emergency departments of hospitals. Additionally, there was unanimous agreement that polypharmacy influences the direct costs associated with migraine treatment (77. 8%).

**Table 3:** Results regarding economic burden of migraine.

Statements	Round	Level of consensus
The overall economic burden of migraine from a societal perspective is estimated to be among the highest along with that of dementia and vascular strokes (57).	1st	77.8% Agreement
The high economic burden of migraine is mainly due to the high loss of productivity of patients (indirect cost) (58, 59).	1st	88.9% Agreement
Migraine patients make extensive use of healthcare resources as they make frequent visits to doctors of various specialties and to the emergency department of hospitals (48, 51, 52, 58–63).	1st	77.8% Agreement
The direct costs associated with migraine treatment are due to polypharmacy (58).	1st	77.8% Agreement

### 3.4 Diagnosis

There was consensus in agreement for eight out of nine statements regarding the diagnosis of migraine (Table 4). Most of medical experts agreed that patients with migraine symptoms might delay seeking medical advice for several years as they learn to live by partially managing their symptoms with simple analgesics. Furthermore, 88.9% of respondents agreed that the stigma and emotional burden experienced by migraine sufferers from their social environment contributes to their hesitance to seek treatment. It was unanimously agreed by all panelists that patients with migraine symptoms are typically referred to other medical specialties before being referred to a neurologist. Also, a consensus was reached (88.9%) that migraine patients often struggle to effectively communicate their symptoms to treating physicians, resulting in delayed diagnosis. In addition, the panel agreed that delay in diagnosis might be partially attributed to the limited amount of time that physicians spend with patients (88.9%) and that the late diagnosis of ΕΜ might cause CM (100%). However, there was no consensus regarding the statement that most migraine patients who visited a doctor in the past year had consulted a neurologist.

**Table 4:** Results on diagnosis.

Statements	Round	Level of Consensus
It may be several years before patients with migraine symptoms seek medical advice, as they have learned to live with their symptoms and manage them partially and ineffectively with simple analgesics (65).	1st	100% Agreement
The stigma and emotional burden experienced by migraine patients from the social environment reinforces their reluctance to seek treatment (55).	1st	88.9% Agreement
Patients with migraine symptoms are initially referred to other medical specialties before being referred to a specialist neurologist (64).	1st	100% Agreement
Migraine patients are not trained to communicate their symptoms to the treating physician, which delays the diagnosis of the disease.	1st	88.9% Agreement
Late diagnosis of migraine may be due to the reduced amount of time the treating physician spends with the patient (27).	1st	88.9% Agreement
Late diagnosis of episodic migraine can lead to progression to chronic migraine (27).	1st	100% Agreement
The majority of migraine patients who have visited a doctor in the last year have visited a neurologist (19).	3rd	72% Neither Agree nor Disagree 28% Disagree

### 3.5 Treatment

Consensus in agreement was reached for 12 out of 15 statements regarding treatment of migraine (Table 5). According to the medical experts, migraine attacks are typically treated with simple analgesics or anti-inflammatory medications, followed by triptans (100%) and 88.9% of respondents agreed that migraineurs frequently overuse medications. A unanimous consensus was reached by the panel that treatment decisions should be based on a patient’s clinical profile, preferences, lifestyle characteristics, and comorbidities (100%). Moreover, they concurred that many migraine patients fail to respond adequately to available treatments or suffer from intolerable adverse reactions (100%), and that a change in treatment could be made even within the same class of medicines in cases of non-response to treatment or adverse effects (77.8%). A consensus in agreement was also reached regarding the statement that preventive treatment should be initiated if the frequency of migraine attacks exceeds 4 days per month for more than 3 months (77.8%). All members of the panel agreed that preventing EM from progressing to CM is one of the most significant benefits of preventive treatment (100%). A consensus of agreement was reached regarding the statement that most patients using non-prescription analgesic treatments for migraine attacks were dissatisfied with the results (100%). Furthermore, the panel reached agreement that many migraine patients who have tried preventive treatment with antiepileptics, antidepressants, or beta-blockers have discontinued the treatment due to unsatisfactory results. In addition, medical experts agreed that patients’ expectations of immediate improvement in symptoms may lead to preventive medications discontinuation prior to their expected improvement (77.8%). Last but not least, the consensus panel agreed that ineffective patient-physician communication is associated with patients’ dissatisfaction with their treatment and poor treatment adherence (100%). However, consensus was not reached for the statement that migraine patients are more likely to use opioid analgesics than the general population (14% Agree, 72% Neither Agree nor Disagree). Additionally, medical experts have not reached a consensus regarding patients’ preferences for the route of administration when it comes to preventive treatments, whether oral or injectable (57% Agree, 43% Neither Agree nor Disagree). Finally, no consensus was achieved regarding the low adherence of patients receiving preventive treatment (57% Agree, 43% Neither Agree nor Disagree).

**Table 5:** Results regarding treatment of migraine.

Statements	Round	Level of Consensus
To manage a migraine attack usually simple analgesics, or anti-inflammatories are used, followed by triptans (38, 39).	1st	100% Agreement
Migraine patients use opioid analgesics more frequently than the general population (81).	3rd	14% Agree 72% Neither Agree nor Disagree 14% Disagree
Migraine patients often overuse medications (66–68).	1st	88.9% Agreement
The treatment decision should be made considering the clinical profile (frequency and intensity of migraines), preferences, lifestyle characteristics and comorbidities of the patients (38).	1st	100% Agreement
Many migraine patients respond inadequately to available treatments or suffer from intolerable adverse events (38).	1st	100% Agreement
If there is no response to treatment by the patient and/or there are adverse events then a treatment change could be made even within the same class of medicines (38).	1st	77.8% Agreement
When the number of migraine attacks exceeds 4 days/month for more than 3 months then preventative treatment is given (38).	1st	77.8% Agreement
One of the important benefits of preventive treatment is to avoid episodic migraine from progressing to chronic migraine.	1st	100% Agreement
The majority of patients using non-prescription analgesic treatments for migraine attacks are not satisfied with the outcome (39).	1st	100% Agreement
Most migraine patients who have tried preventive treatment with antiepileptics, antidepressants or beta-blockers have discontinued the treatment without being satisfied with the results (39).	1st	88.9% Agreement
Migraine patients prefer as a preventive treatment per os treatments to injections (40).	3rd	57% Agree 43% Neither Agree nor Disagree
Migraine patients receiving preventive treatment have low adherence to treatment (69).	3rd	57% Agree 43% Neither Agree nor Disagree
The patients’ improvement may cause them to discontinue prophylactic medication early (69).	2nd	77.8% Agreement
The expectation of migraine patients to immediately show improvement in their symptoms leads patients to early discontinuation of preventive medication (69).	1st	77.8% Agreement
Ineffective patient-physician communication (as perceived and reported by migraine patients) is associated with patient dissatisfaction with their treatment by physicians and poor patient adherence with treatment (56).	1st	77.8% Agreement

### 3.6 Unmet medical need

All statements related to unmet medical needs in migraine management were unanimously accepted (Table 6). It was agreed that healthcare professionals should receive training in the correct diagnosis and treatment of EM to ensure proper disease management (100%). Additionally, consensus was reached regarding the unsatisfactory management of EM despite the wide availability of treatment (88.9%), and that EM should be targeted for prevention, in accordance with its pathophysiology (100%). It was also unanimously agreed that it is imperative to reduce the amount of medication administered to patients suffering from EM during both acute and preventive treatment (100%) and that there is a need for a new treatment for the management of acute migraine without contraindication in patients with cardiovascular problems (100%). Also, the panel noted the importance of simplifying the treatment regimen for EM, suggesting that there is a need for a single medication that could effectively treat migraine attacks and prevent future attacks (88.9%). Finally, the experts agreed that using aids such as apps, patient decision aids, and electronic diaries by migraine patients, enabling them to record crucial information about their migraines and share it with clinicians during their appointments, would enhance patient-physicians communication (88.9%).

**Table 6:** Results regarding unmet medical need of migraine.

Statements	Round	Level of Consensus
Training of HCPs in the correct diagnosis and treatment of episodic migraine is required in order to achieve proper management of the disease (70).	1st	100% Agreement
Despite the wide availability of treatments, the management of episodic migraine remains unsatisfactory (67, 82, 83).	1st	88.9% Agreement
There is a need for targeted preventive treatment of episodic migraine, related to its pathophysiology.	1st	100% Agreement
There is a need for a new treatment for acute migraine that can be safely administered to patients with cardiovascular problems.	1st	100% Agreement
There is a need for treatments whose therapeutic effect in a migraine attack can last for the duration of the attack.	1st	100% Agreement
There is a need for new, safe and targeted treatment for immidiate relief of migraine attack.	1st	100% Agreement
There is a need to reduce the amount of medication a patient with episodic migraine receives during acute and prophylactic treatment.	1st	88.9% Agreement
Simplifying the treatment regimen for episodic migraine with a single drug that can be used both as preventive treatment and for the treatment of migraine attacks would have a positive impact on patient adherence to treatment.	1st	88.9% Agreement
The use of aids (apps, patient decision aids, app, e-diaries) by migraine patients in which they can easily record critical information about their migraine and share it with the clinician during the visit would facilitate communication between them (71).	1st	88.9% Agreement

## 4 Discussion

This study aimed to capture medical experts’ perspective in Greece on burden of migraine and unmet medical needs regarding clinical diagnosis and management. Previous studies have quantified patients’ preferences and satisfaction with treatment as well as the burden of disease in Greece (7, 19, 25, 26, 38–41). To the best of our knowledge, this is the first study to describe Greek medical experts’ point of view through a qualitative analysis. In a recent survey among Greek neurologists on the familiarity and likeability of management and treatment of various neurological disorders, migraine scored high, indicating that physicians like managing migraine and feel confident with its treatment (42).

All statements regarding migraine prevalence, including the higher prevalence in women and among those under 50 years of age, were supported by medical experts, indicating that the existing data are consistent with their clinical experience (5, 7, 9). According to previous studies, migraine is an underdiagnosed disease with various comorbidities occurring in the most productive period of adulthood (5, 43).

In accordance with the literature, it has been agreed that migraine is a burdensome disease for employers, patients, and society in general, as it adversely affects the productivity and social life of patients and their close relatives (10, 11, 38, 44–53). Furthermore, regarding the daily activities of patients our findings are in accordance with a previous Greek study and the international literature (9, 19, 44, 54, 55). In our study, medical experts acknowledged the existence of stigma, as a result of society’s negative perception of the illness and expressed the perception that patients may not feel understood (55, 56).

From an economic perspective, the experts aligned with the international literature and agreed that migraine imposes a substantial financial burden on patients and the healthcare system (57–59). The financial burden is agreed to be attributed to polypharmacy, frequent visits to doctors, and emergency department visits, contributing to increased healthcare resource utilization (48, 51, 52, 58–63).

Our study showed that Greek migraine patients often self-medicate and delay seeking medical help, potentially because of stigma and emotional burden from their environment, in accordance with the literature (55, 64, 65). A major challenge in migraine management is late diagnosis, due to patients’ negligence or to the interference of other physicians before referral to a headache specialist (64). The limited time that physicians dedicate to patients seems to contribute to delayed diagnosis, since migraine is primarily diagnosed clinically (27). As a result, both patients and healthcare providers should become aware of the importance of accelerating the diagnostic process.

The majority of statements related to the clinical management of migraine have been validated by medical experts with their consensus in agreement and were aligned with the available literature (38, 39, 66–68). Furthermore, patients’ expectations of immediate treatment results, ineffective patient-physician communication, and discontinuation of treatment due to symptom improvement seems to lead to patients’ non-adherence. Although it is suggested that oral treatment is more convenient and increases patient adherence, medical experts were not unanimously in agreement about patients’ preference (40). In a recent survey, most Greek migraine patients favored acute medications in tablet form, except CM patients, who preferred subcutaneous treatment due to more severe and prolonged attacks requiring frequent use of subcutaneous sumatriptan as salvage therapy (41). This variability in expert opinion may be attributed to the subjectivity of patients’ preferences. In addition, medical experts have not reached a consensus regarding the issue of low adherence among patients receiving preventive treatment, although the latter is suggested by the literature (69). This may be attributed to the varied clinical scenarios encountered by each physician, making it challenging to generalize conclusions about adherence in preventive treatment. Alternatively, patients may withhold information about their adherence to their physicians.

Targeted training of healthcare professionals (HCPs) in accurate diagnosis and treatment is of paramount importance for the effective management of the disease (70). Considering the prolonged duration of migraine attacks and the associated therapeutic challenges, there is an urgent need for safer and more effective treatments. In addition, streamlining migraine treatment may facilitate adherence to treatment. The use of assistive technologies such as applications, patient decision aids, and electronic diaries facilitates the systematic recording of vital migraine-related information and improves communication between patients and clinicians (71).

Our study’s limitations are primarily related to the Delphi method (72). Firstly, the small number of panelists and secondly, the lack of opportunity to participate in the development of the statements during the Delphi rounds may have limited the comprehension of those statements. As a result of the limited sample size of the Delphi approach, it is difficult to generalize the results to a broader population. In addition, certain statements focused more on the management of episodic migraineurs and our findings apply primarily to EM. However, this is in accordance with the 7-fold prevalence of EM in the Greek population compared to that of CM. This study provides a starting point for designing and conducting future larger and more focused consensus studies in Greece.

## 5 Conclusion

In summary, this Delphi survey reveals migraine’s multivariate impact in patients’ lives, its burden in humanistic and economic terms, and the challenges associated with timely and effective diagnosis and tolerable and effective treatment. Moreover, our findings highlight the obstacles associated with prompt diagnosis and successful treatment. To address the unresolved migraine needs of sufferers, patient-centered approaches, enhanced training for healthcare providers, tailored therapeutic interventions, and advanced communication platforms are essential. Relevant data should be registered continuously and incorporated into clinical practice and health policy decisions in a timely manner.

## Data Availability

The raw data supporting the conclusions of this article will be made available by the authors, without undue reservation.
