# Corrigendum: Unmet needs in the management of migraine in Greece from the perspective of medical experts: a Delphi consensus

**DOI:** 10.3389/fneur.2025.1595681

**Published:** 2025-04-02

**Authors:** Georgia Kourlaba, Michail Vikelis, Theodoros Karapanayiotides, Argyro Solakidi, Dimitrios Trafalis, Katerina Lioliou, Panagiotis Andriopoulos, Aspasia Panagiotou, Dimos-Dimitrios Mitsikostas

**Affiliations:** ^1^Department of Nursing, Faculty of Health Sciences, University of the Peloponnese, Tripoli, Greece; ^2^Headache Clinic, Mediterraneo Hospital, Glyfada, Greece; ^3^2nd Department of Neurology, Faculty of Health Sciences, School of Medicine, AHEPA University Hospital, Aristotle University of Thessaloniki, Thessaloniki, Greece; ^4^Pfizer Hellas, Athens, Greece; ^5^1st Neurology Department, Eginition Hospital, Medical School, National and Kapodistrian University of Athens, Athens, Greece

**Keywords:** migraine, Delphi consensus, neurologists, burden, unmet needs, Greece

In the published article, there was an error. In the **Acknowledgments** section, the original statement did not fully recognize the contributions of individuals involved in the development of this work.

A correction has been made to **Acknowledgments** section which previous stated:

“We would like to thank Professor Sofia Zyga, an employee of the University of Peloponnese, for her valuable contribution with the development of this manuscript.”

The corrected sentence appears below.

